# Resonance-Paced Breathing Alters Neural Response to Visual Cues: Proof-of-Concept for a Neuroscience-Informed Adjunct to Addiction Treatments

**DOI:** 10.3389/fpsyt.2019.00624

**Published:** 2019-09-05

**Authors:** Marsha E. Bates, Laura M. Lesnewich, Sarah Grace Uhouse, Suril Gohel, Jennifer F. Buckman

**Affiliations:** ^1^Cardiac Neuroscience Laboratory, Department of Kinesiology and Health, Rutgers University-New Brunswick, Piscataway, NJ, United States; ^2^Center of Alcohol Studies, Rutgers University-New Brunswick, Piscataway, NJ, United States; ^3^Cardiac Neuroscience Laboratory, Department of Psychology, Rutgers University-New Brunswick, Piscataway, NJ, United States; ^4^Department of Health Informatics, School of Health Professions, Rutgers University-Newark, Newark, NJ, United States

**Keywords:** alcohol, biofeedback, cardiovascular, neural reactivity, functional magnetic resonance imaging, heart rate variability, respiration, resonance breathing

## Abstract

Conscious attempts to regulate alcohol and drug use are often undermined by automatic attention and arousal processes that are activated in the context of salient cues. Response to these cues involves body and brain signals that are linked via dynamic feedback loops, yet no studies have targeted the cardiovascular system as a potential conduit to alter automatic neural processes that maintain cue salience. This proof-of-concept study examined within-person changes in neural response to parallel but unique sets of visual alcohol-related cues at two points in time: prior to versus following a brief behavioral intervention. The active intervention was resonance breathing, a rhythmical breathing task paced at 0.1 Hz (6 breaths per minute) that helps normalize neurocardiac feedback. The control intervention was a low-demand cognitive task. Functional magnetic resonance imaging (fMRI) was used to assess changes in brain response to the cues presented before (A1) and after (A2) the intervention in 41 emerging adult men and women with varying drinking behaviors. The resonance breathing group exhibited significantly less activation to A2 cues compared with A1 cues in left inferior and superior lateral occipital cortices, right inferior lateral occipital cortex, bilateral occipital pole, and temporal occipital fusiform cortices. This group also showed significantly greater activation to A2 cues compared with A1 cues in medial prefrontal, anterior and posterior cingulate, and precuneus cortices, paracingulate, and lingual gyri. The control group showed no significant changes. Thus, following resonance breathing, activation in brain regions involved in visual processing of cues was reduced, while activation in brain areas implicated in behavioral control, internally directed cognition, and brain–body integration was increased. These findings provide preliminary evidence that manipulation of the cardiovascular system with resonance breathing alters neural activation in a manner theoretically consistent with a dampening of automatic sensory input and strengthening of higher-level cognitive processing.

## Introduction

Moment-to-moment changes in internal states (e.g., cognition, emotion, visceral processes, moods) and environments (e.g., cues, persons) influence decisions to use alcohol and other drugs (1). These dynamic, intra-individual change processes derive from the body’s ability to collect and relay information to the brain about the environment (afferent neural traffic), as well as from the brain’s ability to integrate this information and generate a behavioral response (efferent neural traffic). In other words, behavior is influenced by both body and brain signals that are linked via reflexive and predictive bidirectional feedback (2, 3).

**Figure 1:**
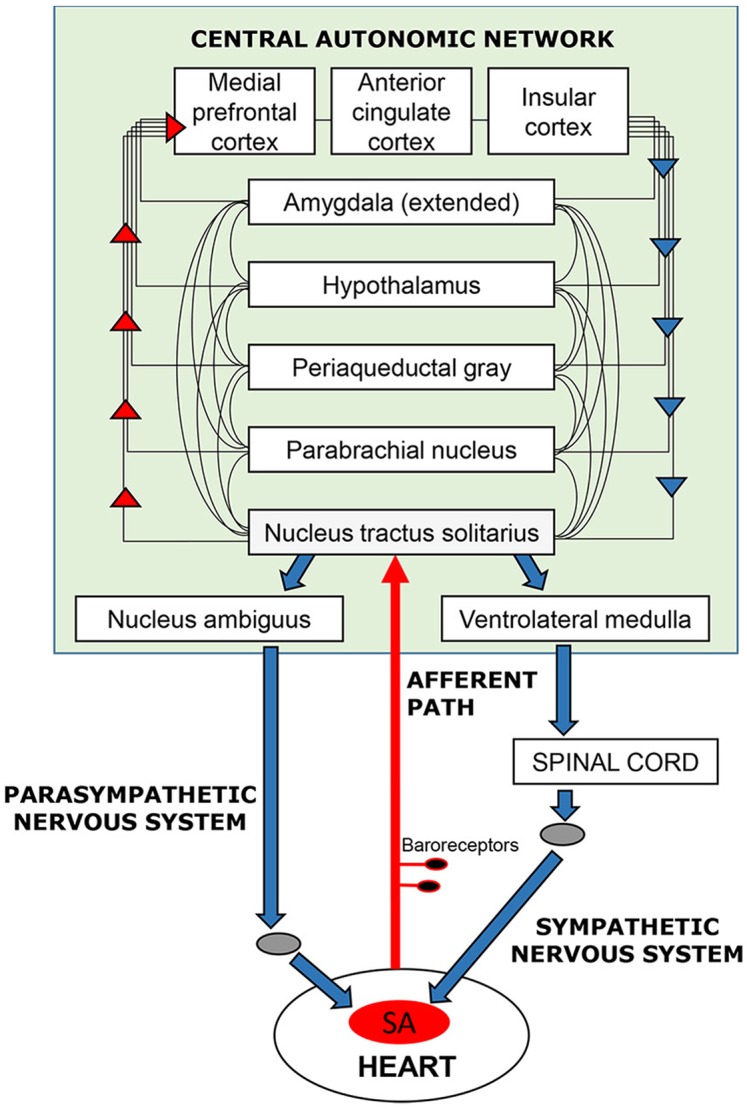
Schematic overview of the neurocardiac feedback loop. Efferent information (blue arrows) emanates from cortical, subcortical, and brain stem structures of the central-autonomic network and flows to the sinoatrial (SA) node of the heart via the sympathetic and parasympathetic branches of the autonomic nervous system. Afferent information (red arrows) from the heart and blood vessels is conveyed back to the brain via baroreceptors located mainly in the walls of the aortic and carotid arteries. Afferent signals enter the brain (shaded in green) via the nucleus tractus solitarius in the brain stem and are integrated with other sensory, cognitive, and affective information as it ascends to cortical regions, including the medial frontal, cingulate, and insular cortices.

In the case of the cardiovascular system, this feedback loop (**Figure 1**) has been extensively documented in terms of its neurophysiology and functional anatomy in rodent and primate models [e.g., (4, 5)]; parallel functional anatomy emerged in a meta-analysis of human neuroimaging studies (6). The loop maintains signaling between the brain and heart via the vagus and sympathetic nerves, baroreceptors located on the aortic arch, carotid artery, and other vessel walls, and a network of brain regions referred to as the central autonomic network (4). These bodies of literature reveal how the brain elicits cardiovascular signals that promote arousal (e.g., increasing heart rate and blood pressure) that, in turn, prepare the organism for goal-directed behavior to respond to in-the-moment demands. Through this loop, feedback from the heart and vasculature is integrated with other autonomic information and relayed to forebrain structures that mediate cognitive and emotional experience (7–9). Consideration of cardiovascular processes as embedded components of affect and cognition implies that these processes contribute to motivated human behavior, including behavioral flexibility toward alcohol and other drugs (10–12). This is important because several non-invasive, low-cost behavioral interventions that help normalize cardiovascular functioning have demonstrated efficacy across various mental and physical health conditions (13–20).

**Figure 2:**
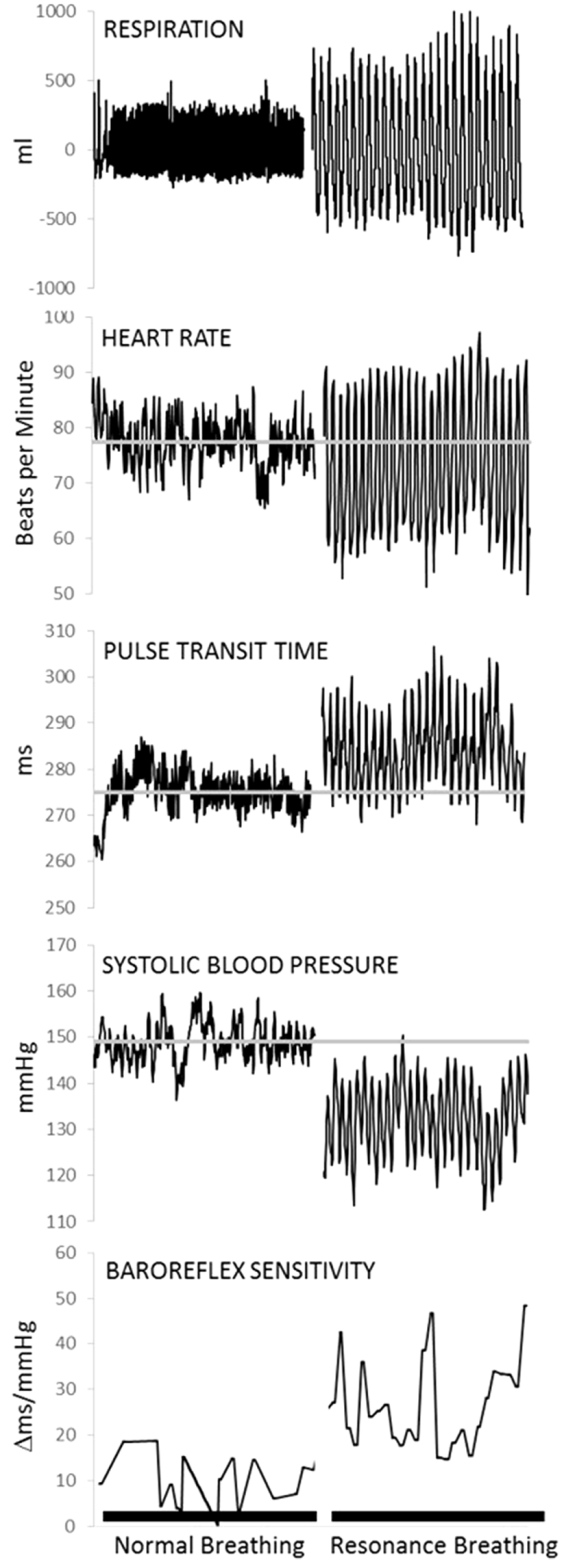
Physiological data from one representative individual collected during a 5-min baseline task (normal breathing) and a 5-min resonance breathing task. Resonance breathing elicited instantaneous changes in respiration, heart rate, pulse transit time (i.e., vascular tone), systolic arterial pressure, and baroreflex sensitivity such that oscillations were magnified and more rhythmic across all measures. In addition, resonance breathing decreased systolic pressure, improved vascular tone, and increased the sensitivity of the neurocardiac feedback loop (i.e., baroreflex). Adapted from (12). Used with permission.

Two compelling qualities of the neurocardiac feedback loop for intervention development are its plasticity and responsivity to relatively simple behavioral interventions. Afferent stream activation of the neurocardiac feedback loop can be accomplished by manipulating peripheral functions, such as respiration and muscle flexion (21–24). Breathing paced at 6 breaths per minute (0.1 Hz) is slower and more rhythmical than typical breathing (12–20 breaths per minute). It creates resonance within the cardiovascular system by synchronizing cardiac oscillations driven by respiratory sinus arrhythmia (i.e., the phenomena of heart rate acceleration with inhalation and deceleration with exhalation) with cardiac oscillations driven by the baroreflex, which links heart rate acceleration/deceleration to corresponding changes in the blood pressure (21, 25). As shown in **Figure 2**, breathing at this frequency lowers systolic blood pressure, increases variability in the time intervals between R-spikes of the electrocardiogram (ECG) (i.e., heart rate variability), generates large oscillations in pulse transit time (i.e., vascular tone variability), and increases the sensitivity of heart rate to changes in blood pressure (i.e., baroreflex gain) (12, 21). A recent meta-analysis found that clinical interventions involving paced breathing at a resonance frequency of the cardiovascular system resulted in large effect size reductions in anxiety and stress (26). Preliminary evidence also suggested paced breathing may reduce craving for appetitive substances (27).

The brain structures of the central autonomic network that participate in cardiovascular signaling overlap considerably with those that process reward, emotion, and habit formation (28), including medial prefrontal, cingulate, and insular cortices, and amygdala. These structures also figure prominently in current translational models of putative addiction neurocircuitry (29–31), with the brain stem serving as the first point of neural integration of afferent autonomic and somatic signals from the body. Psychophysiological evidence suggests that the neurocardiac feedback loop may participate in substance use behaviors through its contribution to attention capture by stimulating cues, affective modulation, and relay of visceral reactivity to the brain [e.g., (32–36)], but little research has extended these findings to the neural structures that comprise the central autonomic network. Nonetheless, converging lines of evidence suggest that ineffective or maladaptive functioning of this feedback loop can set into motion a cascade of biological events that alter one’s ability to adaptively modulate affect, arousal, and stress response (2, 37, 38).

Neural cue reactivity studies, wherein brain activation is measured while participants are exposed to salient alcohol- or drug-related cues, have received significant attention in the neuroscience and psychology of addiction literatures (39, 40). Cue reactivity studies typically compare within-person differences in brain activation to alcohol or drug cues versus control cues (40). There is substantial evidence that elevated alcohol and drug cue neural reactivity is found in individuals with substance use disorders (40–42). Increased neural (43) and cardiac (44) cue reactivity also has been associated with increased drug craving (2, 37, 38). Experimental evidence suggests that heightened neural (45) and cardiovascular (46) reactivity to alcohol and drug cues is related to high risk substance use in non-clinical populations. Thus, altered reactivity to affective and appetitive stimuli appears to increase the likelihood that individuals will be susceptible to contextual influences on substance use, even following extended periods of abstinence (47, 48). This raises the question of whether behavioral interventions that enhance the efficiency of neurocardiac signaling might be used to alter neurocardiac activation to contextual challenges that promote substance use and relapse (49, 50).

This proof-of-concept study examined whether stimulating the afferent stream of the neurocardiac feedback loop with a 5-min course of resonance breathing can affect subsequent neural activation to visual alcohol cues. In contrast to cue reactivity paradigms that compare neural activation to alcohol versus control cues, this study examined within-person changes in neural response to alcohol cues at two points in time. We compared neural activation with unique sets of alcohol cues viewed prior to versus following the breathing task. Because this is the first study of its kind, there is no empirical literature to guide predictions about brain activation changes when participants are exposed to visual cues following resonance breathing. Based on the anatomy of the central autonomic network (4, 5) and drug cue salience networks (28, 40), we hypothesized that significant changes in activation may be observed in brainstem, medial prefrontal, cingulate, and insular cortices, as well as in the amygdala. We further allowed for the possibility of spreading activation, wherein structures within the central autonomic network that share additional network circuitry with regions outside the central autonomic network (e.g., the mesocorticolimbic circuit, ventral striatum) may exhibit activation changes as well. Significant changes in neural response were not anticipated in the group that viewed alcohol cues before and after completing a low-demand cognitive task.

## Methods

### Participants

Forty-nine men and women, ages 18 to 25 years, were recruited at a large, northeast U.S. university and in the surrounding community through advertisements targeting alcohol drinkers. Initial inclusion criteria for all participants assessed via self-report were fluency in English, right-handedness, near 20/20 vision (corrected), and alcohol consumption at least once per month. Exclusion criteria assessed via self-report included: MRI contraindications (e.g., permanent metal in the body, claustrophobia), abnormal hearing, any serious medical condition (e.g., epilepsy, diabetes), cardiovascular problems (e.g., hypertension, heart murmur), current learning disability or attention difficulties, loss of consciousness for longer than 30 min, and, for women, pregnancy. To reduce heterogeneity related to psychiatric comorbidities and poly-substance use, lifetime diagnosis of a bipolar disorder or psychosis (e.g., schizophrenia, schizoaffective disorder), past year psychiatric/psychological treatment, past year cannabis use exceeding four times per month in the past year, other past year illicit drug use more than twice per month, past or current substance use treatment (including Alcoholics Anonymous/Narcotics Anonymous), and substance use during pregnancy on the part of the biological mother also were exclusionary.

Half of the participants were recruited based on meeting the National Institute on Alcohol Abuse and Alcoholism (NIAAA) “low risk” drinking criteria [i.e., no more than 5 drinks per day for men (4 drinks per day for women), no more than 14 drinks per week for men (7 drinks per week for women)], as well as an additional criterion of not binge drinking more than once in the past 6 months. The other half met DSM-IV-TR criteria (51) for alcohol dependence. This proof-of-concept examination of resonance breathing as a neurally active intervention included all participants with the exception that data from eight participants were excluded due to excessive motion in the scanner. The final sample (n = 41) had a mean age of 21.4 (SD = 1.9) years and was racially and ethnically diverse (27% Asian, 27% black/African American, 29% white, 17% other/multiple race; 11% Latino/a); 46% of the participants identified as female.

### Procedures

Potential participants who gave verbal consent completed a telephone screening interview to determine initial eligibility. Eligible participants were asked to abstain from alcohol and drug use (except caffeine and nicotine) for 24 h prior to the experimental session. After screening, they were randomized into the active intervention (i.e., resonance breathing) or the control intervention (i.e., vanilla task), with drinking profiles being approximately equally distributed in both groups.

Upon arrival at the imaging center, participants provided written informed consent, supplied a breath sample to verify zero blood alcohol concentration, and completed a MRI safety screener and self-report questionnaires regarding alcohol use, mood state (Positive and Negative Affect Scale) (52), and stress (Perceived Stress Scale) (53). Basic physiological measures (e.g., temperature, blood pressure, weight) and a urine sample were collected; participants with a positive urine screen for cocaine, methamphetamine, opiates, and/or benzodiazepines (One Step Multi-Drug Screen Test Panel) were excluded. Participants with a positive urine screen for marijuana were asked additional follow-up questions about their drug use, and those with marijuana use exceeding four times per month were excluded. Women were screened for pregnancy using a standard urine dipstick. All participants were trained to use an MRI-compatible response box and to perform their assigned intervention task. Task training lasted approximately 2 min. Participants then were fitted with ECG sensors and a respiration belt and positioned in the scanner.

**Figure 3:**
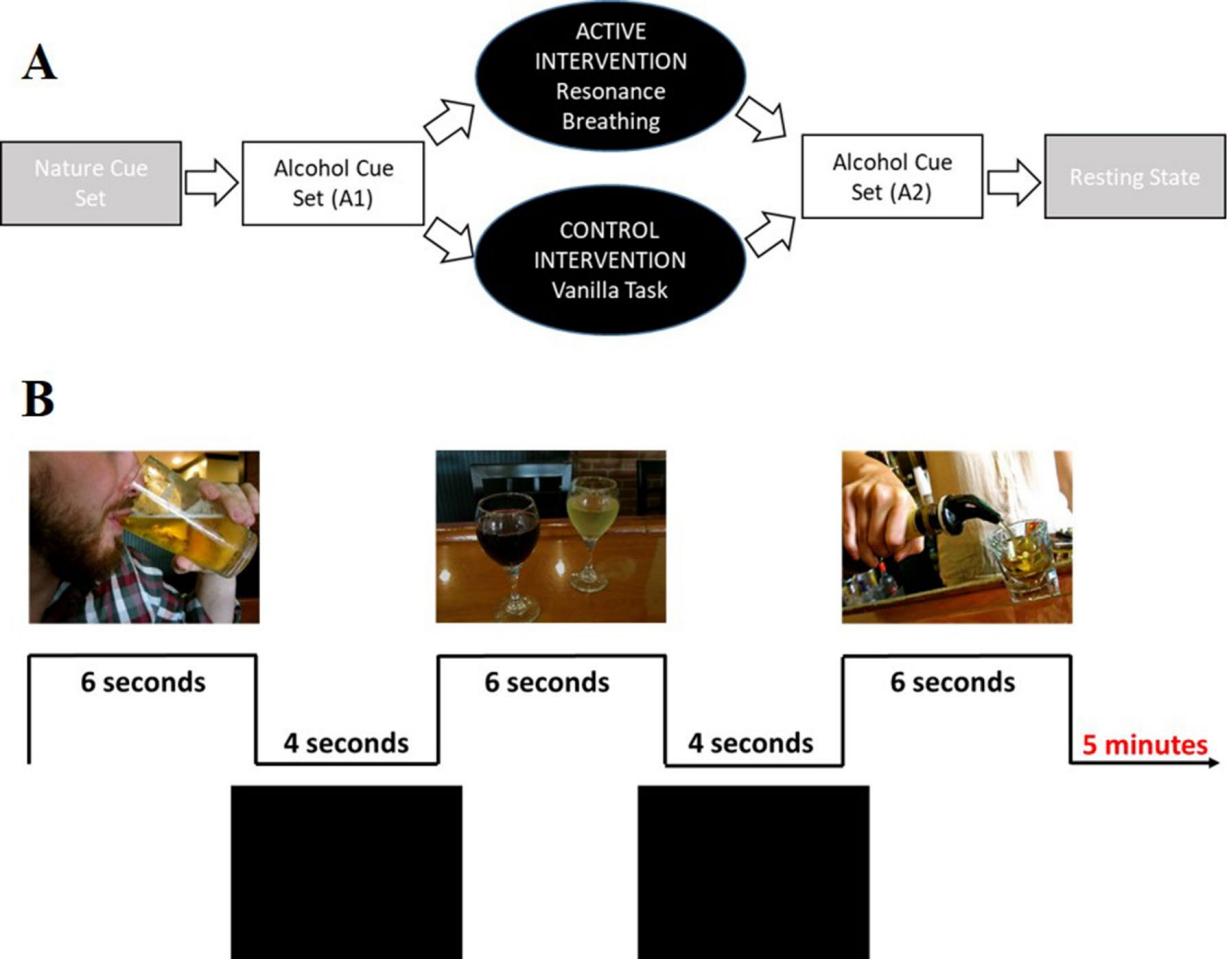
Visual depiction of the study and cue task design. Panel **(A)** shows the complete study design. Participants first viewed a set of nature picture cues (data not shown). Participants then viewed a set of alcohol picture cues (A1), followed by a 5-min intervention task (active condition: resonance breathing; control condition: vanilla task). They then immediately viewed a second, distinct set of alcohol picture cues (A2). The study ended with a 6-min resting state task (data not shown). Panel **(B)** shows representative images from the alcohol cue tasks, both of which involved viewing 30 unique images that were presented for 6 s, with 4-s inter-stimulus intervals.

The overall paradigm (**Figure 3A**) involved four 5-min tasks: 1) viewing a set of nature picture cues, 2) viewing a set of alcohol picture cues (A1), 3) performing the intervention task, 4) viewing a second, distinct set of alcohol picture cues (A2); a 6-min resting state task was then performed. After each task, participants responded to the question, “How much are you currently craving alcohol right now?” using a track ball on a visual analogue scale (VAS, 43) anchored from “not at all” (0) to “extremely” (100). Stimulus cues were presented using E-Prime software (Psychology Software Tools Inc.). Images were projected onto a screen positioned at the rear of the scanner bore and viewed through a mirror attached to the head coil. A trigger pulse synchronized the start of each task with the E-Prime software. Total scan time was approximately 45 min.

Data from the two alcohol cue tasks (A1, A2) were analyzed in the present study. Each task included 30 unique images that were presented for 6 s with 4-s inter-stimulus intervals (**Figure 3B**), a design driven by the larger study’s broader goal of characterizing the relationship between cardiovascular and neural reactivity. The alcohol cues were drawn from prior studies in our and others’ laboratories (34, 54, 55). Each participant’s self-reported preferred beverage (i.e., beer, wine, “straight” liquor, or mixed drinks) made up approximately 50% of the images to which they were exposed. Participants were instructed to pay attention to the images and to press a response box button when they saw an image that contained their preferred drink.

Between the A1 and A2 cue sets, participants in the active intervention (resonance breathing) synchronized their breathing with a visual pacer at the rate of 0.1 Hz (i.e., 6 breaths per minute). Compliance to the breathing task was verified via analysis of the respiratory signal. Time series respiratory frequency data were Fourier transformed, and the shape of the spectrum was visually inspected; all participants showed a respiratory peak at 0.1 Hz and spectral characteristics consistent with resonance breathing. Participants in the control intervention group completed a low-demand cognitive “vanilla” task wherein different colored rectangles were presented for 10 s each; they were instructed to silently count the number of blue rectangles (56).

After exiting the scanner, participants were compensated for their time. Those who met the criteria for alcohol dependence were given an informational brochure on alcohol use disorders and treatment options. This study was approved by the university’s institutional review board for the protection of human subjects involved in research.

### Imaging Parameters and Pre-Processing

Imaging data were collected using a 3T Siemens Trio scanner and 12-channel head coil. Standard localizer, anatomical, scout, and field map scans were collected. High-resolution anatomical images were acquired using a T1-weighted MPRAGE protocol with parameters: repetition time (TR) = 1,900 ms, echo time (TE) = 2.51 ms, matrix = 256 × 256 voxels, field-of-view (FOV) = 256 mm, voxel size = 1 × 1 × 1 mm, 176 1-mm sagittal slices (.5 mm gap). Functional blood oxygen level-dependent (BOLD) data were acquired using single-shot gradient echo-planar imaging (EPI) sequences with parameters: TR = 2,000 ms, TE = 25 ms, flip, angle = 90°, matrix = 64 × 64 voxels, FOV = 192 mm, voxel size = 3 × 3 × 3 mm, 35 contiguous 3-mm sagittal slices (1 mm gap). ECG and respiration data were collected using a MRI-compatible BIOPAC acquisition system (Biopac Systems, Goleta, CA) as part of the larger study.

FSL 5.0.9 software was used to conduct image preprocessing and data analysis (FMIRB’s Software Library, https://fsl.fmrib.ox.ac.uk). Non-brain tissue was removed from all anatomical and BOLD images using FSL’s Brain Extraction Tool (BET, 57) by estimating each image’s center of gravity and manually adjusting BET parameters as necessary until an optimal result was obtained. BOLD data were motion-corrected using FSL’s MCFLIRT (58), and the output was reviewed to identify participants with excessive motion during the resting-state scan. Excessive motion was defined conservatively as mean absolute and/or relative displacement greater than .5 mm. A paired t-test was performed to compare mean framewise displacement between the randomized intervention groups. No significant differences were observed in motion between the groups (*p* > 0.05). BOLD images were segmented into gray matter, white matter (WM), and cerebral spinal fluid (CSF) using FSL’s FAST (59). Probability maps of CSF and WM were derived, and time-series data for these signals were extracted from each participant. These nuisance parameters (i.e., WM, CSF) along with extended head motion parameters were used as covariates in the linear regression models implemented in FSL to decrease the effects of signals-of-no-interest. BOLD data were registered to standard space with a two-step process using FMRIB’s Linear Image Registration Tool (FLIRT) (60). The data were first registered to the T1-weighted anatomical image and then to MNI-152 standard space using 9 degrees-of-freedom and SINC interpolation. All data were visually inspected for gross errors in registration. A high pass temporal filter was set to 50 s, and spatial smoothing was set to a 6-mm full-width at half-maximum Gaussian kernel.

### Statistical Analyses

Analyses of the BOLD data from the A1 and A2 cue reactivity tasks were performed using a two-step process. Subject-level effects were calculated using first-level analyses in FSL’s FEAT, and group effects were determined using higher-level analyses. In the first-level analysis, each alcohol image event was modeled and convolved with a double-gamma hemodynamic response function (HRF), and the mean task activation for A1 and A2 was calculated for each participant. In the higher-level analysis stage, two sets of analyses were performed using Randomise, the non-parametric permutation-testing tool implemented in FSL (61). First, one-sample *t*-tests were conducted to characterize neural activation in each intervention group before (A1) and after (A2) the intervention. Next, to examine intervention effects on neural activation to visual stimuli, paired *t*-tests with two contrasts were conducted on each intervention group (i.e., resonance breathing, control) separately (61). For each contrast, 5,000 permutations were calculated. One contrast (A1 > A2) was designed to determine brain areas that demonstrated greater activation pre- compared to post-intervention task, and the second contrast (A2 > A1) was designed to determine brain areas that demonstrated greater activation post-intervention compared to pre-intervention task. Threshold-free cluster enhancement was employed (62), and activation was considered significant at *p* < 0.05 (corrected for multiple comparisons using FSL Randomise).

A repeated-measures mixed model was used to assess the effect of resonance breathing on VAS craving scores. Craving data for one participant was missing due to equipment failure; thus, data from 40 participants were available for analysis. A between-subjects factor of intervention group (resonance breathing, control) and a within-subjects factor of craving scores following A1 and A2, as well as their interaction, were modeled. To examine the relationship between VAS craving scores and brain regions that exhibited significant pre-intervention to post-intervention changes, regions-of-interest (ROIs) were defined by creating 6-mm spheres around the peak voxel of each significant cluster of activation for the A1 > A2 and A2 > A1 contrasts. Mean activation values of these ROIs were extracted for each participant from the subject-level A1 and A2 cope images. Pearson correlations were then used to test the associations between ROI activation and VAS craving scores at A1 and A2. Point biserial correlations were used to examine the relationship of binary drinking status (low-risk = 0, alcohol dependent = 1) to ROI activation at A2 in the resonance breathing group. These analyses were performed using SAS 9.4 software (SAS Institute, Cary, NC, USA).

## Results

### Neuroimaging

**Figure 4:**
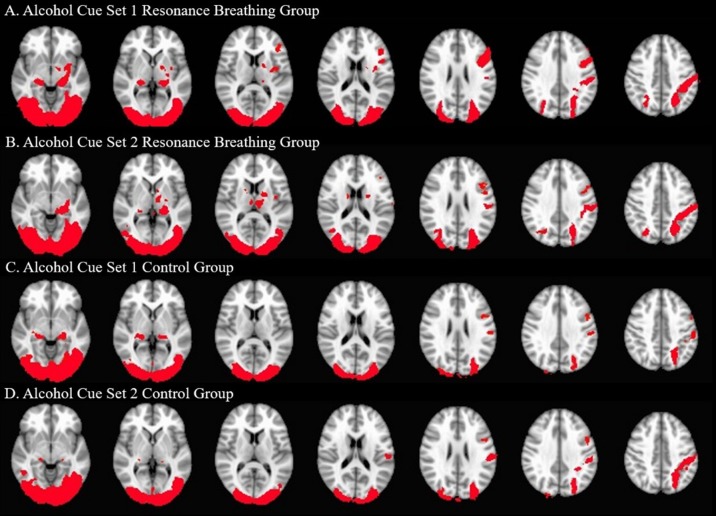
Significant Neural Activation to Visual Alcohol Cue Sets. One-sample *t*-tests were used to identify areas of significant neural activation during alcohol cue set viewing. The neural responses of the active intervention (resonance breathing) group are shown in Panels **(A)** (A1 task, cues viewed prior to the intervention) and **(B)** (A2 task, cues viewed after the intervention). The neural responses of the control intervention (vanilla task) group are shown in Panels **(C)** (A1 task, cues viewed prior to the intervention) and **(D)** (A2 task, cues viewed after the intervention). Axial slices are shown in MNI standard space at z = −6 (first slice) and every fourth subsequent slice. Images are oriented using radiological convention. Areas of significant activation are shown in red.

Both intervention groups exhibited widespread neural activity in response to the visual alcohol cues, including in bilateral posterior parahippocampal gyri, temporal occipital fusiform cortices, lateral occipital cortices (inferior and superior divisions), postcentral gyri, and cerebellum at A1 and A2. The resonance breathing group (n = 22) additionally showed significant activation in bilateral inferior frontal gyri, left insula, left pallidum, left putamen, left amygdala, and left thalamus (A1, A2), and left precentral gyrus (A1). The control group (n = 19) additionally showed significant activation in the right thalamus (A1) and left precentral gyrus (A2). These results are shown in **Figure 4**.

**Figure 5:**
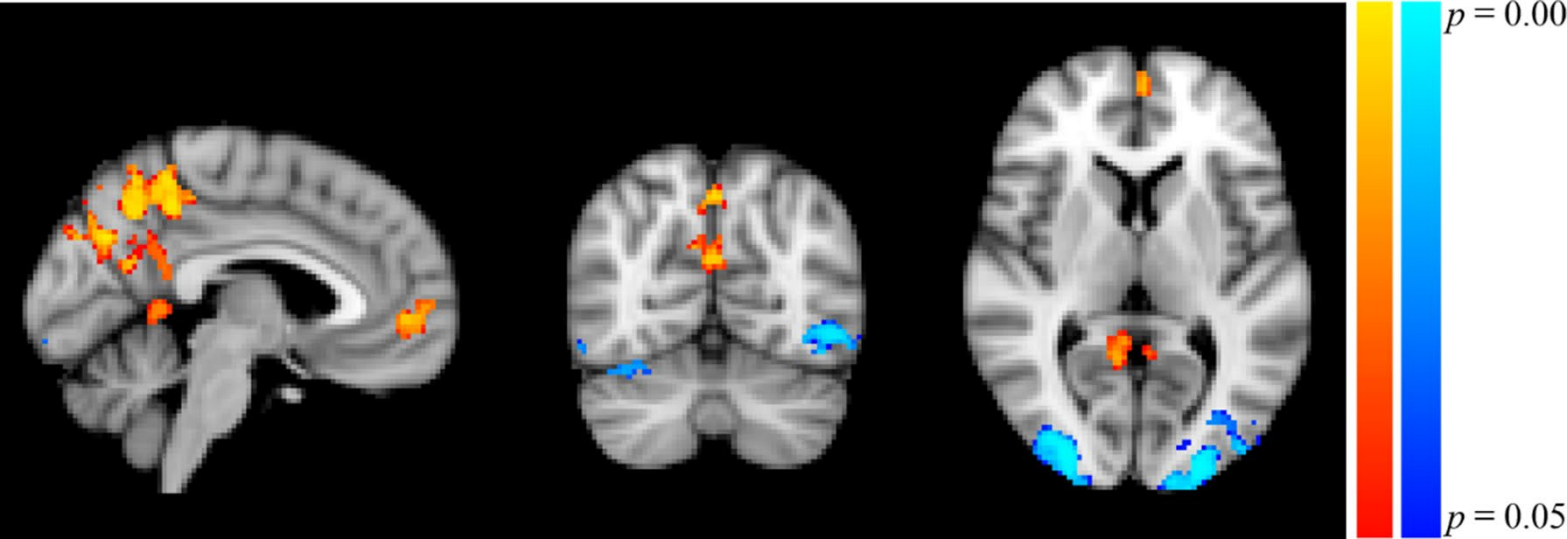
Significant Clusters of Activation in Resonance Breathing Group. Blue-cyan clusters represent regions with greater activation during A1 compared to A2 (A1 > A2), and red-yellow clusters represent regions with greater activation during A2 compared with A1 (A2 > A1). Voxels were thresholded at *p* < 0.05. Image is shown in MNI standard space at x = −4, y = −66, z = 6, and oriented using radiological convention.

Participants in the resonance breathing group demonstrated greater activation in response to alcohol cues pre-breathing compared with post-breathing (A1>A2) in left inferior and superior lateral occipital cortices and right inferior lateral occipital cortex, as well as bilateral occipital pole and temporal occipital fusiform cortices. They also demonstrated greater activation post-breathing compared with pre-breathing (A2 > A1) in voxels spanning precuneus cortex, posterior cingulate gyrus, and bilateral lingual gyri, as well as in medial prefrontal cortex (MPFC), paracingulate gyrus, and anterior cingulate cortex (ACC). These results are shown in **Table 1** and **Figure 5**.

**Table 1:** Anatomical location at peak voxel coordinates in significant clusters of activation in resonance breathing group.

A1>A2 Contrast					
		MNI Coordinates			
Cluster Size	Z	x	y	z	Peak Voxel Anatomical Location
2,433	6.80	−30	−96	10	Occipital pole (L)
1,339	6.95	28	−90	4	Occipital pole (R)
10	4.74	28	−38	−24	Temporal fusiform cortex (R)
A2>A1 Contrast					
		MNI Coordinates			
Cluster Size	Z	x	y	z	Peak Voxel Anatomical Location
2,141	7.81	2	−78	42	Precuneus Cortex
283	5.89	−2	50	0	Paracingulate Gyrus/Medial Prefrontal Cortex
150	4.74	−16	−50	−2	Lingual Gyrus (L)
16	5.35	6	−24	40	Posterior Cingulate Cortex
15	4.44	2	−22	32	Posterior Cingulate Cortex
2	3.81	0	−56	10	Precuneus Cortex
1	6.87	−2	−6	36	Anterior Cingulate Cortex

The control group analysis yielded no significant activation in either the A1 > A2 or A2 > A1 contrasts, indicating that there were no statistically significant changes in brain activation in response to visual alcohol cues in the group that performed the control task.

### Self-Report

Surveys administered prior to the neuroimaging session revealed that the sample as a whole had low-moderate perceived stress (mean ± standard deviation = 17 ± 6), and positive (mean ± standard deviation = 30.7±9.7) and negative (mean ± standard deviation = 13.9 ± 6.2) affect scores that were similar to those reported from the original general adult normative sample (52). There were no differences in affect or stress between the intervention groups nor between the drinking groups (all *p* > .05).

Craving was measured in the scanner after exposure to each cue block. The results of a repeated-measures (A1, A2) mixed model indicated that there was a significant main effect of group on craving, but no main effect of task (i.e., from pre- to post-intervention). Participants randomized to the resonance breathing intervention group reported lower levels of in-the-moment craving compared to those randomized to the control intervention group [*F*(76,1) = 5.76, *p* = 0.0188; least square mean ± standard error of resonance breathing group = 28.1 ± 4.5 and of control group = 43.7 ± 4.7]. The group–task interaction was not statistically significant, suggesting that changes in subjective reports of craving pre- to post-intervention did not significantly differ between the two groups.

No significant correlations were observed at A1 between VAS craving scores and the ten cluster activation scores in the full sample (*r* range, −0.24 to 0.09, all *p* > .05). In addition, there were no significant correlations at A2 between craving scores and the ten cluster activation scores within either group (resonance breathing group *r* range, −0.32 to 0.28, all *p* >.05; control group *r* range, −0.42 to 0.44, all *p* > .05). Lastly, there were no significant correlations at A2 between drinking status (AUD vs. low risk) and the ten-cluster activation scores in the resonance breathing group (*r* range, −0.27 to 0.28, all *p* > .05).

## Discussion

Evidence that visceral afferent signaling influences stimulus processing argues for intervention development aimed at manipulating cardiovascular signals to alter detection and neural processing of affective stimuli (63). The results of the present study provide the first proof-of-concept evidence that a brief behavioral intervention of resonance breathing can significantly alter drinkers’ neural activation to visual alcohol cues. The observed changes in brain activity included both decreases and increases in the activation of distinct brain regions.

In the group that performed resonance breathing between the visual cue tasks, but not in the control group, there was reduced activation in occipital regions from the first set of alcohol cues to the second, different set of alcohol cues. This pattern of results suggests that the breathing intervention prompted a subsequent decrease in visual cortex activation when individuals were confronted with alcohol-related visual stimuli. The specificity of these changes to alcohol-related content is unclear as this proof-of-concept study did not include a cue set of non-alcohol–related images presented before and after the intervention. Indeed, visual cortex activation to many types of images, including faces, is modulated by their emotional and social significance (64–66). Multiple lines of evidence also support the involvement of the visual cortex in appetitive cue processing. Several meta-analyses found that drug users consistently showed increased activation in occipital regions in response to drug-related cues compared to controls, even when non-visual drug-related stimuli were presented (41, 67–69). Increased visual cortex activation has been observed in individuals with behavioral addictions, such as pathological gambling, as well (70–72). Thus, although the literature suggests that the role of the visual cortex in alcohol and drug cue reactivity is not specific, decreased activation in the lateral occipital cortices following resonance breathing would be consistent with decreased perception, representation, and recognition of the images (73) and/or may potentially reflect less attention being directed toward the cues by the amygdala (66) or higher cortical areas (74).

In parallel with reduced visual processing of the cues, we observed increased activation in bilateral medial prefrontal, anterior and posterior cingulate, and precuneus cortices during the second alcohol cue task, only in the resonance breathing group. The ACC and MPFC, as regions of the central autonomic network, bi-directionally influence, and are influenced by, afferent cardiovascular signaling. Resonance breathing increases cardiovascular input to the brain via activation of brainstem nuclei that share connectivity with the ACC and MPFC (4) and are thought to give rise to the visceral experience of emotion (75). Functionally, the ACC is a part of the mesocorticolimbic circuit, which is thought to be involved in conflict monitoring and the regulation of cognitive and emotional processing by integrating input and modulating processing in other regions (76, 77). The MPFC is considered to be part of a cognitive control system in the brain that promotes goal-directed behaviors (78) by using incoming information to predict the most adaptive response based on past experience (79).

Hypothetically, increased activation of MPFC and ACC in response to alcohol cues following the breathing intervention would be consistent with heightened internal monitoring of cognitive-emotional state and enhanced cognitive control. At the same time, some studies have identified these regions as sites of heightened reactivity to alcohol and other drug cues (42), and heightened reactivity in these regions has been related to post-treatment drinking and relapse, although the results in this area have not been consistent (80). Thus, it is unclear whether or under what circumstances these and other brain regions accentuate or restrain cue-elicited craving and substance use behaviors. Evidence for individual differences in brain areas most reactive to appetitive cues (42) and inconsistencies in replication add further complication to interpretation. More nuanced examination of intra-individual changes in neural activation across brain areas, and perhaps also across simultaneously operating psychological and physiological systems involved in motivated behavior, are needed.

The posterior cingulate cortex (PCC) and the precuneus showed increased activation to visual alcohol cues following the breathing intervention, but not the control task. Both of these regions are considered core nodes of the default mode network, a functional brain network involved in self-referential thought and mind-wandering (81) that shows preserved connectivity during cognitive load (82). The lingual gyrus, a brain region involved in visual encoding and higher-order analysis of complex visual stimuli (83), also showed increased activation only in the resonance breathing group. This gyrus has been implicated in spontaneous thought and often co-activates with the default mode network (84). Whether increased activation in these regions potentially plays a role in promoting self-regulation in response to alcohol or other affectively valenced cues is unknown, but warrants further investigation. One possibility is that following resonance breathing the brain reverts to its “baseline” resting state (85) for some amount of time despite activation by salient cues, rather than transitioning to a heightened state of arousal.

We did not observe acute changes in self-reported craving levels in the resonance breathing group following the second presentation of alcohol cues (absence of significant cue task by group interaction), nor were craving levels related to brain clusters of activation in response to cues at A1 or A2. Several factors likely contributed to these null findings. Randomization into resonance breathing and control groups in the present study did not result in equivalent mean craving rating scores; the resonance breathing group reported significantly lower craving levels throughout the study. Failures of randomization in small samples are common (86), and future studies may benefit from selecting participants with high levels of self-reported craving and/or matching on craving levels across intervention groups. It may also be that the brief 5-min duration of resonance breathing did not affect conscious self-estimates of craving in the present sample, or that resonance breathing works in a way that affects a different pathway, such as the operation of cue salience (50), rather than consciously experienced craving levels. The present data are limited in not speaking to these alternative speculations.

### Implications for Clinical Translation

If replicated and extended, the current findings that a brief, 5-min bout of resonance breathing changed neural activation in brain areas implicated in affective and appetitive stimulus processing could have clinical implications for individuals who show elevated neural reactivity in response to appetitive cues (44). Resonance breathing is the active mechanism of heart rate variability biofeedback, an empirically supported behavioral intervention for disorders with core features of affective and emotional dysregulation (13, 14, 17, 26) including alcohol use disorders (44, 87, 88). Emerging evidence suggests that heart rate variability biofeedback and paced breathing interventions reduce self-reported craving for alcohol and other appetitive stimuli, such as food (27, 89). While standard heart rate variability biofeedback delivery protocols include five to ten 1-h sessions and home practice (90, 91), resonance breathing itself produces immediate physiological effects (see **Figure 2**). This proof-of-concept study was novel in examining whether resonance breathing also elicits immediate neural effects. The findings provide an initial step in validating resonance breathing as an in-the-moment behavioral tool that potentially could be used ad lib in the natural environment to alter neural activation, both before and during contexts of heightened risk for substance use. Accessible smart phone applications are available to self-administer resonance breathing and HRV biofeedback, suggesting promise for a scalable intervention tool if future research is successful in demonstrating that such effects are linked to reduced alcohol and drug use behaviors.

### Limitations and Directions for Future Research

As a proof-of-concept study, these findings should be interpreted with caution and used for the generation of future hypotheses regarding the effects of resonance breathing on neural activation to alcohol-related visual stimuli, behavioral correlates of alcohol use such as in-the-moment craving, and actual use behaviors. Importantly, the changes observed in neural activation to the cues following the resonance breathing intervention should not be considered specific to alcohol-cue reactivity, as this study did not include a comparison condition of matched, non-alcohol cues presented before and after the intervention. This study also was limited in not being sufficiently powered to examine sensitively the relation of individual differences in alcohol use behaviors to changes in neural activation following resonance breathing. We note that the cue presentation paradigm of the present study was designed in line with the goal of better understanding afferent cardiovascular input to neural reactivity and thus was not typical of those used in many other fMRI studies of cue reactivity. A recent meta-analysis found that cue paradigm and type did not significantly influence neural response patterns associated with cue reactivity however (28), suggesting the fMRI assessment of neural activation is robust to multiple cue presentation approaches. Future studies should include larger samples to link current and chronic substance use behaviors to cue reactivity, and a design that counterbalances and compares neural response to alcohol-related and non-alcoholic beverage cues. Specificity may be addressed also by comparisons to non-alcohol or drug-related, yet positive or negative affectively valenced, visual cue sets.

### Conclusion

In summary, this study presents preliminary evidence that individuals ranging in drinking behaviors from low-risk to alcohol-dependent may be less visually engaged by alcohol cues and initiate greater top-down cognitive processing of cues following resonance breathing. This is consistent with the broader literature on resonance breathing that shows it normalizes neurocardiac feedback and improves autonomic nervous system regulation (25). Moreover, it points to a potential neural foundation for the effects of resonance breathing and adds to the scientific premise for the use of heart rate variability biofeedback as an intervention for brain-based mental and physical health conditions. More highly powered studies are needed to replicate and extend these neural activation results. Critical next steps are to understand how the cardiovascular and neural changes elicited by resonance breathing are linked to changes in the subjective experience of craving and alcohol use behaviors.

## Ethics Statement

This study was carried out in accordance with the recommendations of the National Institutes of Health guidelines for ethical treatment of human subjects with written informed consent from all subjects. All subjects gave written informed consent in accordance with the Declaration of Helsinki. The protocol was approved by the Rutgers University Arts and Sciences Institutional Review Board for the Protection of Human Subjects Involved in Research.

## Author Contributions

MB and JB designed the study. MB and LL wrote the first draft of the article. All authors contributed to writing sections of the manuscript. LL and SU collected all data. LL and SU post-processed the imaging data. LL, SU, and SG performed the data analyses. All authors read and approved the final manuscript.

## Funding

This work was supported in part by R21AA022748 and K24AA021778 (MEB), K02AA025123 (JFB), and F31AA027147 (LML) from the US National Institutes of Health.

## Conflict of Interest Statement

The authors declare that the research was conducted in the absence of any commercial or financial relationships that could be construed as a potential conflict of interest.
